# A Design of Partial Textured Surface on Gear Washers for Reducing Friction and Wear under Low Speed and Heavy Load Conditions

**DOI:** 10.3390/ma14164666

**Published:** 2021-08-19

**Authors:** Yang Liu, Hui Zhang, Guangneng Dong

**Affiliations:** Key Laboratory of Education Ministry for Modern Design and Rotor-Bearing System, Xi’an Jiaotong University, Xi’an 710049, China; lyly000@stu.xjtu.edu.cn (Y.L.); donggn@xjtu.edu.cn (G.D.)

**Keywords:** surface textures, friction, wear, distribution, gear washer

## Abstract

This paper presents the effort to reduce friction and wear of gear washers under low-speed and heavy-load conditions by designing the arrangement of surface textures. The influence of distributional parameters of textures on load-bearing capacity and friction coefficient of gear washers are studied numerically to obtain a preferable surface texturing design. Then, experimental tests were carried out to plot the Stribeck curves of the obtained texture arrangement compared with bare surface and another unoptimizable texture distribution arrangement to facilitate the verification of the simulation results. Theoretical predictions illustrate that the annular gear washers with partial surface texturing provide lower friction coefficients than bare washers. Textures having a sector angle of 20°, a coverage angle of 12°, a circumferential number of 8, and a radial number of 6 are selected as the final optimal surface texture distribution design. Experimental results confirm that the obtained texture arrangement moves the Stribeck curve towards the lower left, indicating thickening of oil film thickness and reduction in friction coefficient. In addition, the weight loss caused by wear is also reduced by the optimized texture design.

## 1. Introduction

A gear washer is a mechanical component in an annular shape coupling with the gear’s back surface to form a friction pair, protecting the gearbox from scuffing and damage [1]. Generally, a gear washer is operated under the condition of low speed and heavy load. The lubrication regime is mixed lubrication, in which the contact of asperities occurs [2]. Such severe lubrication conditions may result in friction, wear, damage, and even failure of the friction pair. Moreover, the wear and damage of washers bring in variation and temperature rise of the friction pair, and it may aggravate the friction and wear recursively. Thus, it is of great significance to reduce friction and wear of gear washers to prolong the maintenance period and the lifespan of the gearbox, especially in heavy-duty machinery (such as lorries and loading machines, et al.).

As a simple, economical, and effective method for improving tribological performance, surface texturing, involving fabricating micro-structures, such as dimples, grooves, and protruding pits, on the frictional interface through physical or chemical methods, has been studied theoretically and experimentally by many research groups [2,3]. Their results have revealed that a suitable geometrical shape and arrangement of surface textures can significantly increase the hydrodynamic load-bearing capacity of the friction interface to avoid the direct contact of asperities between counter-mating parts, thus reducing the friction coefficient and wear, enhancing the reliability and prolonging service lifespan. Typical examples of successful application of surface texturing can be seen in the fields of slider bearings [4], piston cylinder systems [5], mechanical seals [6], and others [7,8]. Shen Gang et al. [9] fabricated micro-groove textures on a cobalt–chromium–molybdenum material combination of bioimplants, and demonstrated that micro-groove textures could optimize tribological performance, showing a reduction of 51.9% compared to that of polished samples without micro-grooves. Chang Tie et al. [10] designed three kinds of new Koch snowflake textures on the surface of the UHMWPE to decrease friction and prolong the service life of the bearings. However, it is also demonstrated that unsuitable design of distribution and geometrical parameters of surface texturing may bring in little improvement or an unfavorable effect on their tribological performance [11]. Subsequently, to guarantee the positive effect of surface texturing design, many researchers focused on investigating the influence of geometrical and distributional parameters of surface textures and elucidating corresponding mechanisms.

Due to the wide application of surface texture on many different mechanical fields, various geometrical shapes of surface texture have also been extensively studied. As micro-circular pits are easy to fabricate and give relatively good tribological characteristics [12,13], they were popularly adopted as the surface textures on mechanical components by many researchers. Studies provided a great number of optimal circular texture parameters for the specific friction pairs under given operating conditions. Typically, Zhang et al. [4] arranged circular dimples in rectangular and linear radiate arrays on Babbitt alloy disks. Their experimental results demonstrated that textures in a square array with an area density of 8.6% yield the lowest friction coefficient. Yan [11] evaluated experimentally in an attempt to identify the significance of dimple parameters. Results suggested that dimples, with an area ratio of 5%, depth range of 5–10 μm, and diameter between 100 and 200 μm, could actually induce a friction reduction as high as 77.6% compared to that of untextured surfaces. As shown in the above studies, a textured ratio of 5–15%, depth/diameter ratio in the range of 0.01–0.05, and diameter of 100–1000 μm are generally identified as preferable geometrical parameters of circular textures [4,11,14].

Besides geometrical shapes and parameters, the distribution arrangement of textures on the surface of mechanical friction pairs also influences tribological behaviors. Square (grid-like), linear radiate, and staggered arrays are typical texture layouts arranged on mechanical friction interfaces [4,15]. Researchers investigated the tribological influence of these typical texture distributions. Typically, the work of Hua et al. [15,16] shows that circular dimples arranged in rectangular and linear radiate arrays on disks demonstrated that the former arrays were equipped with a lower and more stable friction coefficients in a rotary sliding motion. Zhan and Yang [17] studied the effectiveness of distribution angles (the angle between dimples) of the surface texture for a cylinder liner-piston ring system. They found that the way of distributing texture could influence the wear characters of the tribological system, and the effect of wear reduction was most significant when its angle was 60°. Lu and Liu [18] derived a method by designing and fabricating lubricating dimples of a sliding bearing with a phyllotactic pattern. Recently, Zhang et al. optimized the distribution of circular concave textures on sectorial thrust bearing pads to improve the load-bearing capacity and reduce the friction coefficient [19]. Zhao et al. discussed the influence of the texture position of circular pits on the friction force of sliding bearings. Xie et al. [20] enhanced tribological properties of GCr15 samples with optimal density and depth of micro-textures. Their experimental tests confirmed the significant tribological improvement of such a pattern. Zhang et al. [21] optimized the coverage of circular micro-textures on bearing sliders, aiming at improving their tribological performance.

The above studies have discussed the influence of the distribution of surface textures for their specific material and operating conditions theoretically and experimentally. Moreover, some researchers have focused on applying and optimizing the distribution of surface texture in engineering fields, such as bearings, by integrating theoretical and experimental methods. Zhang et al. [22] proposed an effective oil layer method for improving the processing quality and the tribological behavior of a laser-textured surface. Zhang et al. [23] explored the distribution of circular dimples on journal bearings, theoretically based on hydrodynamic pressure effect, and proposed their favorable arrangement. Marian [24] theoretically analyzed a thrust bearing with square textures partially covering its inlet, and the simulation results were compared with experiments conducted in a test rig. Their effort facilitates the obtainment of a set of optimization parameters of surface texturing. Rahmani et al. [25] investigated the effects of partial-texture location and depth on tribological performances of journal bearings using a computational fluid dynamics (CFD) method, based on the Navier–Stokes equation. They reported that shallow textures at the inlet region of a journal bearing could significantly improve journal bearings’ tribological behaviors. Liu et al. [26] derived an analytical solution to pressure for hydrodynamic lubrication problems encountered in the fan-shaped step bearing, which can be useful for maximizing bearing performance. In addition, in the study of Henry et al. [27], the textures on thrust bearings can reduce friction up to 30% at low loads. The above studies [23,24,25] present the possible application of optimized surface texturing on slider bearings or thrust bearings, and give preferable distribution/layout arrangements for their specific operating conditions. In addition, the kind of partial radiation array texture distribution [24,26,27] was put forward by researchers to improve the tribological properties of slider bearings.

However, there is little existing research that focuses on the application of surface texture on gear washers under the operating condition of low speed and heavy load. So, this research aims to reduce friction and wear of gear washers by arranging surface texture distribution/layout properly on its surface. The favorable distribution/layout arrangement with proper geometrical parameters was firstly determined based on the simulation results of a mixed lubrication model. Then, Stribeck curves and weight loss caused by wear of the obtained textured surfaces were plotted experimentally to compare with the bare surface, confirming the superior tribological performance of the surface texturing arrangements obtained in the study.

## 2. Mixed Lubrication Model

The mixed lubrication model was employed for numerical analysis and following simulations, which combined the average flow Reynolds equation and the asperity contact model. The software MATLAB was used to investigate the influence of surface texture on tribological properties, and then to determine the design of the surface texture.

For the gear-washer friction unit, since it generally operates under the severe conditions of low speed and heavy load, the film thickness between frictional interfaces is relatively small; thus, direct contact of asperities occurs to share part of the load with hydrodynamic pressure—this is the so-called mixed lubrication. In this case, both the micro-hydrodynamic action and contact of asperities influence the tribological behaviors [3]. Textures on the surface of mechanical components may form convergent channel gaps so that the filled lubricant can act as Rayleigh bearings to generate micro-hydrodynamic pressure, thus thickening film thickness and reducing friction/wear.

In this study, allowing for the effect of asperities on hydrodynamic pressure, the average flow Reynolds equation (in cylindrical co-ordinates) proposed by Patir and Chen [28] is employed as the governing equation to evaluate the hydrodynamic uplift of the textured surface, as Equation (1):

(1) $$\frac{\partial }{\partial r}(\varphi_{x}h^{3}r\frac{\partial p}{\partial r})+\frac{1}{r}\frac{\partial }{\partial \theta }(\varphi_{y}h^{3}\frac{\partial p}{\partial \theta })=6\varphi_{c}r\omega \eta \frac{\partial h}{\partial \theta }+6r\omega \eta \sigma \frac{\partial \varphi_{s}}{\partial \theta },$$

where *p* is the average lubrication film pressure; *θ* is the angular co-ordinate; *r* is the radial co-ordinate; *R*_0_ is the external radius of the washer, *σ* is the effective roughness; *η* is the viscosity of the lubricant; *φ_x_* and *φ_y_* are the pressure flow factor in the circumferential and radial direction, respectively; *φ_c_* is a contact factor proposed by Wu [29], while *φ_s_* is a shear flow factor; *ω* is the angular velocity; *p_a_* is the atmospheric pressure; *p_c_* is the cavitation pressure; and *h*_0_ is the minimum film thickness.

At the same time, the classical Greenwood [30] asperity contact model is used to compute the contact pressure in the present study. In the model mentioned above, the heights of asperities are assumed to obey the Gaussian distribution and only elastic deformation occurs. The average contact pressure is expressed as Equation (2):

(2) $$p_{c}=K^{\prime }E^{\prime }F_{2.5}(\lambda ),$$

in which, *E*′ is the effective elastic modulus ($\frac{1}{E^{\prime }}=\frac{1-v_{1}^{2}}{2E_{1}}+\frac{1-v_{2}^{2}}{2E_{2}}$), *E*_1_ and *E*_2_ are the elastic modulus of the materials of the friction counterparts, respectively, (*E*_1_ is assumed to be 90 GPa for a bronze washer, while *E*_2_ is preset as 200 GPa for a steel gear); *v*_1_, *v*_2_ are the Poisson’s ratios of the materials of the friction counterparts, which are both assumed to be 0.3; referring to the study of Hu [31], *K*′ is assumed to be 5.318748 × 10^10^ × *σ*^2.5^; and *F*_2.5_(λ) is the function related to the distribution of the asperity heights.

In addition, in such a mixed lubrication regime, due to the combination of the average flow Reynolds equation and the asperity contact model, the tribological performance is usually determined by both the hydrodynamic effect and the contact of asperities. The load-bearing capacity *W_c_* and friction *F_c_* in a contact regime are expressed as Equations (3) and (4), respectively:

(3) $$W_{c}=\int \int_{\Omega }p_{c}dxdy,$$

(4) $$F_{c}=W_{c}\mu_{c},$$

where *μ_c_* is the friction coefficient between asperities on contact surfaces in a boundary lubrication regime. For a bronze–steel friction pair, it is assumed to be 0.2.

The load-bearing capacity *W_f_* and friction *F_f_* in a hydrodynamic regime can be derived as Equations (5) and (6), respectively:

(5) $$W_{f}=\int \int_{\Omega }pdxdy,$$

(6) $$F_{f}=\int \int_{\Omega }\tau dxdy,$$

where *τ* is the shear stress of fluid.

Subsequently, the total load-bearing capacity *W* and friction *F* could be derived by summing up their corresponding values in contact and hydrodynamic regimes, as shown in Equations (7) and (8), respectively:

(7) $$W=W_{c}+W_{f},$$

(8) $$F=F_{c}+F_{f},$$

**Figure 1:**
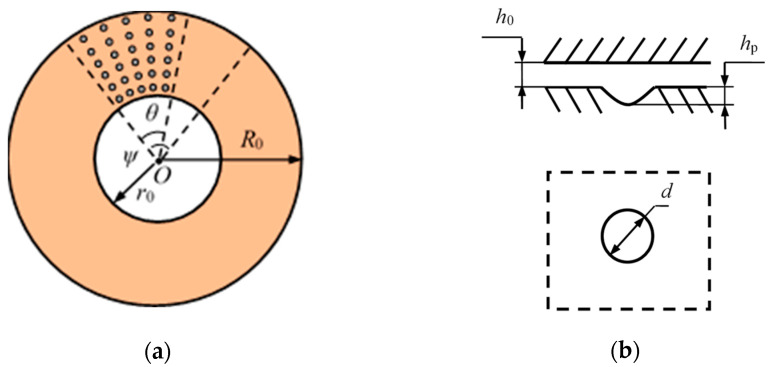
Illustration of (**a**) distribution and (**b**) geometrical parameters of surface textures in the study.

In the integral equation above, as the relative rotational movement repeats the tribological condition of an annular sector of the washer periodically, the annular sector is selected as the computation domain. A periodic condition described as a function of the sector angle *ψ* (Figure 1) is thus specifically imposed on the lateral boundaries of the computational domain, as shown in Equation (9):

(9) p(0)=p(ψ),

Meanwhile, geometry of the gap between the upper smooth surface and the lower textured surface may divide the lubrication film into (i) a liquid phase and (ii) a cavitation phase where liquid evaporation is taking place. In the cavitation region (denoted in terms of angular co-ordinate Ω), employment of the Reynolds boundary condition with pressure distribution satisfying Equation (10) can thus guarantee the computational efficiency.

(10) $$p(\Omega )=p_{c},\frac{\partial p(\Omega )}{\partial \theta }=0$$

## 3. Theoretical Predictions

### 3.1. Parameters Preassigned

The annular shape of a gear washer is illustrated in Figure 1a. Circular pits are fully or partially distributed on the annular gear washer. For textures partially covering the washer, the angle of sector is defined as the angle of a periodical sector of partial-textured surface, as shown in Figure 1, in which *θ_c_* is the coverage angle of textures. The geometrical parameters of a dimple are presented in Figure 1b, in which *h_p_* is the depth and d is the diameter.

In this section, the geometrical and operational parameters are presented as: outer radius *R*_0_ = 66 mm; inner radius *r*_0_ = 45.5 mm; dimple depth *h_p_* = 8 μm; dimple diameter *d* = 1000 μm; roughness of the counterparts *σ*_1_ = *σ*_2_= 0.8 μm; effective roughness *σ* = 1.13 μm; minimum film thickness *h*_0_ = 2 μm, rotational speed *n* = 10 rpm; and viscosity of lubricant *η* = 20 mm^2^/s (100 °C).

### 3.2. Types of Texture Arrays

**Figure 2:**
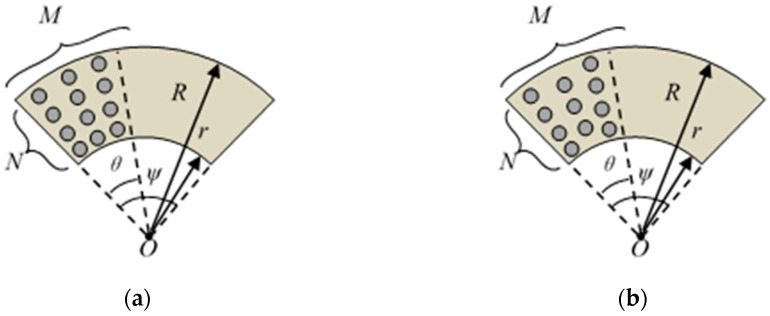
Geometrical parameters of textures in (**a**) radiation array and (**b**) staggered radiation array.

After the basic parameters were determined, four typical texture distributions, radiation array and staggered radiation array [24,26,27] (as shown in Figure 2), fully or partially covering the annular surface, are involved in this section. They are marked as: A—partial radiation array, B—partial staggered radiation array, C—full radiation array, and D—full staggered radiation array. The parameters (Figure 2) of texture arrangements A and B are presented as: *ψ* = 60°, *θ* = 30°, *N* = 10, *M* = 15. The parameters (Figure 2) of texture arrangements C and D are presented as: *ψ* = 60°, *θ* = 60°, *N* = 10, *M* = 30.

**Figure 3:**
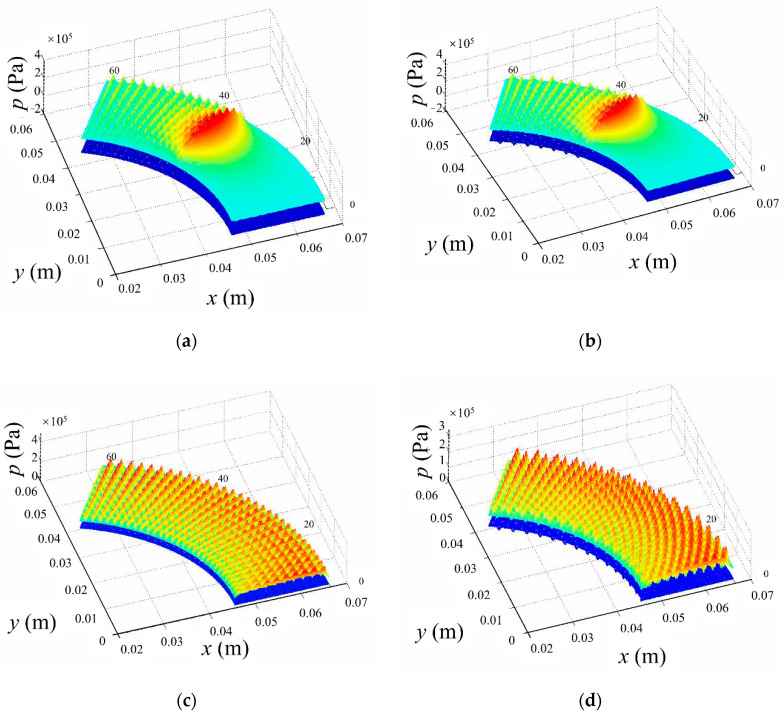
The pressure distribution of four typical texture distribution arrangements: (**a**) A—partial radiation array; (**b**) B—partial staggered radiation array; (**c**) C—full radiation array and (**d**) D—full staggered radiation array.

**Figure 4:**
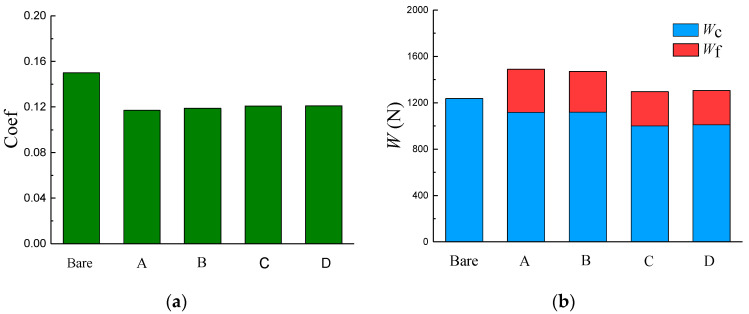
(**a**) Friction coefficient and (**b**) load-bearing capacity of the four typical texture arrangements.

Figure 3 illustrates the pressure distribution of the four typical texture arrangements (arrangement A–D). There exist small local pressure peaks for all textured washers (arrangement A–D). These small local pressure peaks seem to have a one-to-one correlation with textures. Moreover, an obvious hydrodynamic pressure peak can be observed for partial texture arrangements (arrangement A and B), which implies higher hydrodynamic pressure and load-bearing capacity. The predicted contact/hydrodynamic load-bearing capacity and friction coefficient of the four surface-texturing arrangements are shown in Figure 4. Partial texture arrangements give relatively higher hydrodynamic load-bearing capacity and lower friction coefficient than full texture arrangements, which agrees with the pressure distribution described in Figure 3. Texture arrangements A and B seem to have a similar load-bearing capacity and friction coefficient. However, staggered texture distribution seems to be more uniform than rectangular distribution, which may bring in more uniform contact pressure and secondary lubrication action [16]. Consequently, in the present study, the staggered partial texture distribution is selected as the distribution form for the following study. The subsequent optimization process is based on the conclusions in this section.

### 3.3. Influence of Distributional Parameters

In quick succession, for a better elaboration of the influence of distribution, four parameters, *N_t_*, *K*, *K_m_*, *K_n_*, are specifically defined in this study. *N_t_* is defined as the number of periodical sectors dividing the annular uniformly, which has a correlation with the angle of sector *ψ*, i.e., *N_t_* = 360°/*ψ*. *K* is the ratio of *θ_c_*, the coverage angle of textures, to *ψ* (*K = θ/ψ*). The two parameters, *K_m_* and *K_n_*, reflecting the density of textures in circumferential and radial directions, are stipulated as: *K_m_ = Md / (r · θ)*, *K_n_ =Nd / (R - r)*, respectively.

**Figure 5:**
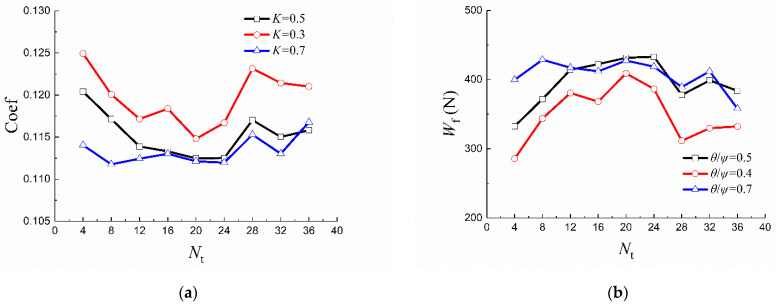
(**a**) Friction coefficient and (**b**) hydrodynamic load-bearing capacity varying with *N_t_*.

Figure 5 shows the friction coefficient and hydrodynamic load-bearing capacity, varying with *N_t_* from 4 to 36 with an interval of 4. It is obviously observed that, when *N_t_* is in the range of 16–24, the friction coefficient curve exhibits relatively low values, while the hydrodynamic load bearing capacity curve exhibits relatively high values.

**Figure 6:**
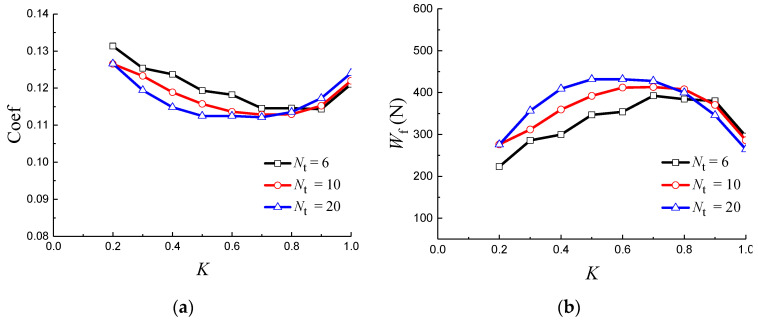
(**a**) Friction coefficient and (**b**) hydrodynamic load-bearing capacity varying with *K*.

The friction coefficient and hydrodynamic load-bearing capacity varying with *K* are presented in Figure 6. When *K* is in the range of 0.6 to 0.7, the friction coefficient and hydrodynamic load-bearing capacity have their minimum and maximum values, respectively.

**Figure 7:**
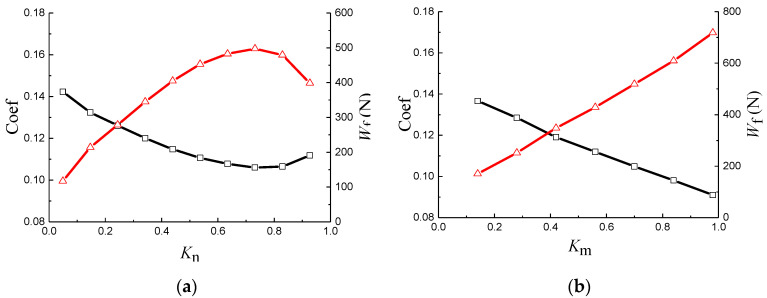
Friction coefficient and hydrodynamic load-bearing capacity, varying with (**a**) *K_n_* and (**b**) *K_m_*.

Figure 7a,b illustrates the changing trend of friction coefficient and load-bearing capacity with *K_n_* and *K_m_*, respectively. As exhibited in Figure 7a, the friction coefficient seems to decrease initially with the increase in *K_n_*, then, after that, it presents an increasing trend. When *K_n_* is in the range of 0.6–0.8 (*N* = 10–16), the friction coefficient gives its minimum value. The hydrodynamic load-bearing capacity has a varying inverse trend with the Coef-*K_n_* curve. Unlike *K_n_*, friction coefficient and hydrodynamic load-bearing capacity have an almost linear functional correlation with the parameter *K_m_* (Figure 7b). A higher *K_m_* results in a relatively higher hydrodynamic load-bearing capacity and lower friction coefficient.

### 3.4. Determination of Distributional Parameters

As previously mentioned, the favorable coverage area ratio of textures on a metal surface proposed by most researchers is about 5–15% [4,11,12,14,32,33], which may restrict the number of dimples in circumferential and radial directions, i.e., *M* and *N*. The area of a periodical vector can be derived as *S = πK(R − r*^2^*)/N_t_*, while the coverage area of textures can be calculated as *S_t_* = *π(MN*-*M*/2)*d*^2^/4. According to Section 3.3, *N_t_* and *K* are identified to be 18 and 0.6, respectively. Referring to the favorable coverage area ratio, 5% ≤ *S_t_/S* ≤ 15%, the restriction formula can be expressed as: 16 ≤ *M*(*N −* 0.5) ≤ 45. As *N* should be in the range 10–16, the possible *M* and *N*, and their corresponding coverage area ratio of textures are tabulated in Table 1. The lighter green-colored table cells are the feasible *M* and *N* that met the proposed coverage area ratio 5–15% [4,11,12,14,32,33]. Analysis on Figure 7b demonstrates that a higher *M* may bring in better tribological behavior for surface texturing. Consequently, the value of *M* is identified to be 8, and, thereby, *N* is selected to be 6, as shown in the deeper green-colored textbox in the table. Consequently, the final geometrical parameters of the texture distribution on the gear washer are determined to be: *N_t_* = 18; *K* = 0.6; *N* = 6; *M* = 8.

**Figure 8:**
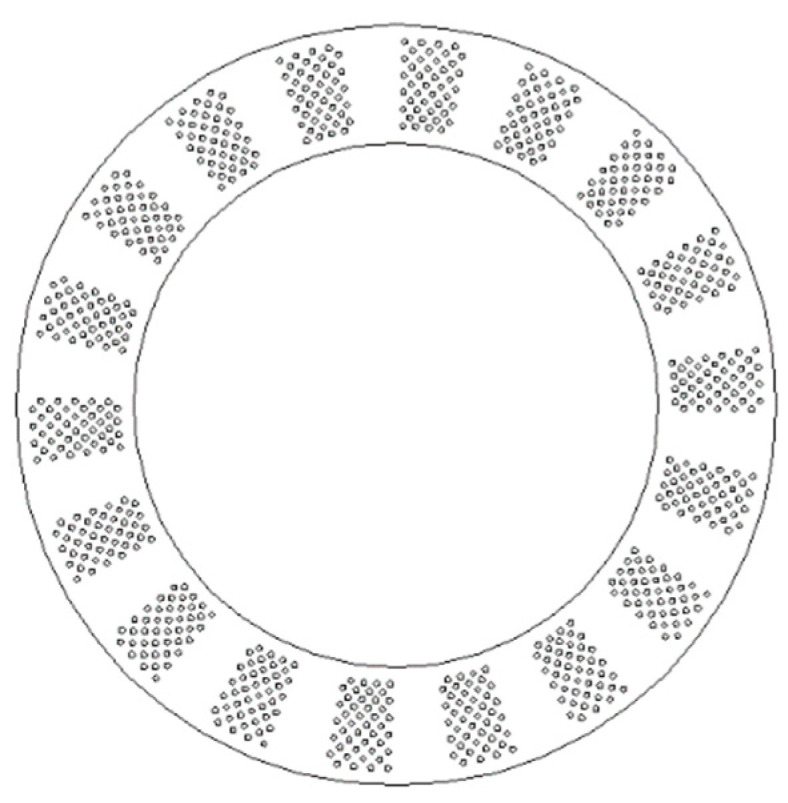
Distribution of texture with distributional parameters: *N_t_* = 18; *K* = 0.6; *N* = 6; *M* = 8.

The favorable annular washer with the obtained texture distribution is plotted in Figure 8, in which the number of periodical sectors is 18, the coverage angle of textures to the angle of sector is 0.6, the number of dimples in circumferential directions is 8, and in radial directions 6.

## 4. Experimental Verification

### 4.1. Tribological Experimental Tests

**Figure 9:**
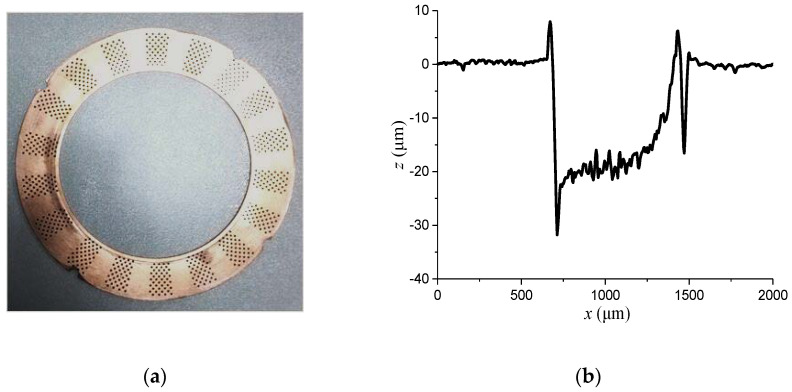
(**a**) Photograph of a textured gear washer and (**b**) longitudinal section of a dimple.

To confirm the favorable tribological performance of the obtained surface texturing, a corresponding test rig was set up to plot the Stribeck curve of the bare and textured gear washers. The bare surface of a tin bronze washer mating with the back surface of 45 steel of a half-axle gear forms a friction pair. The roughness and hardness of the bare surface of a gear washer are about 0.8 μm and HB 120, respectively. For the mating gear, its back surface has a roughness of 0.8 μm and a hardness of HRC 60. Surface textures were manufactured on the surface of the gear washer through an LSF20II laser marking machine, HGLASER Corporation, China. In addition to the textures with geometrical parameters (*N_t_* = 18; *K* = 0.6; *N* = 6; *M* = 8) determined in the simulation section (marked as Texture-2), a textured washer, with geometrical parameters of *N_t_* = 12; *K* = 0.6; *N* = 6; *M* = 5, is also involved in this section as a comparison (marked as Texture-1). Figure 9 shows the photograph of a textured gear washer and the longitudinal section of a dimple. According to Figure 9b, it can be estimated that the average depth of a dimple is about 20 μm.

**Figure 10:**
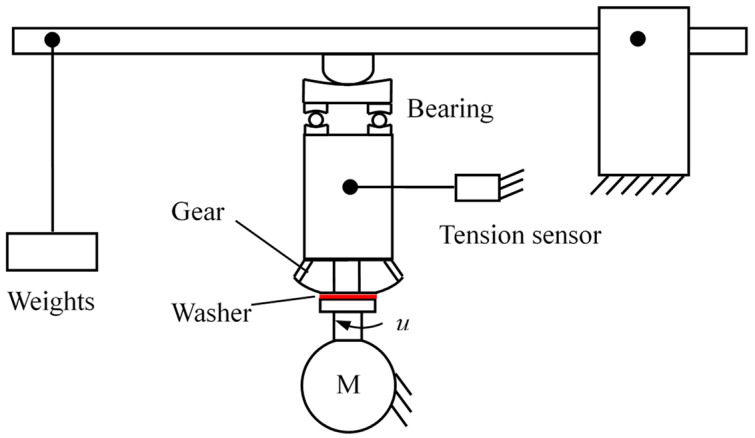
Schematic of the washer test rig.

The schematic of the test rig for the washer-to-gear system is shown in Figure 10. The washer is driven by a motor to rotate under the stationary gear, which is balanced by a line connected to a tension sensor to record the friction simultaneously during the test. A load is applied to the washer-to-gear friction pair by using a lever with weights, as shown in Figure 10. The lubricant involved in the present study is heavy-load lubrication oil for vehicle gears (QL-5 85W-90, Kunlun lubricant of China National Petroleum Corporation), which has a viscosity of *η* = 20 mm^2^/s (100 °C). Before mounting the washer on the rotational platform, the washers were cleaned with acetone for 10 min in an ultrasonic cleaner, and then were dried with a blower. Then, the lubricant is supplied to the disk by the BT50-1J peristaltic pump. In the present study, the load applied to the friction pair was 590 N, 805 N, 1020 N, 1236 N, 1450 N, and the rotation speed of the washer was preset as 240 rpm, 270 rpm, 300 rpm, 330 rpm, 420 rpm, 510 rpm, 600 rpm. After 7 h running-in, friction coefficients of each test were obtained by averaging its values during a test period of 3 min. The testing temperature and the relative humidity are approximately 25 °C and 60%, respectively.

### 4.2. Results and Discussions

**Figure 11:**
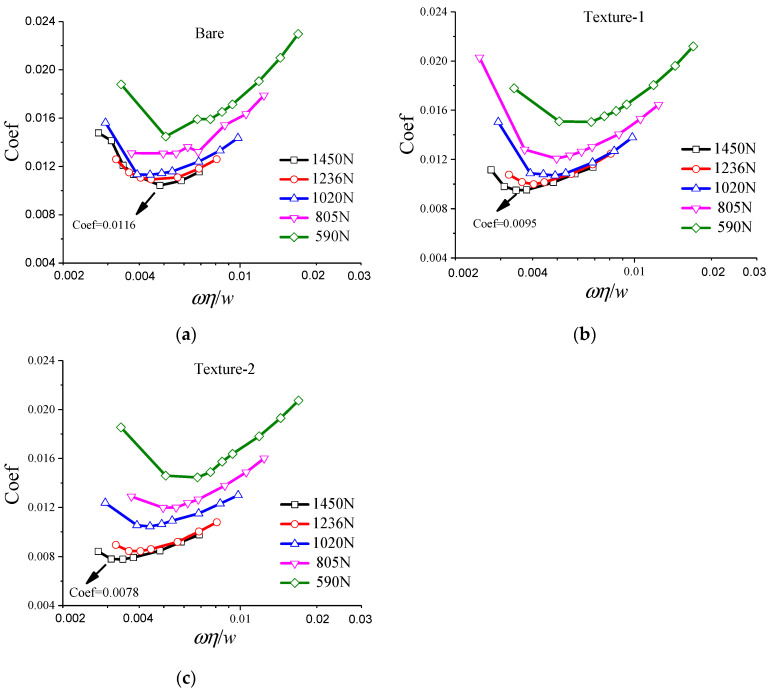
Stribeck curve of (**a**) bare, (**b**) textured-1, and (**c**) textured-2 gear washers.

The Stribeck curves of gear washers with bare, textured-1 (*N_t_* = 12; *K* = 0.6; *N* = 6; *M* = 5) and textured-2 (*N_t_* = 18; *K* = 0.6; *N* = 6; *M* = 8) surfaces are plotted in Figure 11a–c, respectively. It is observed that the two textured surfaces (textured-1 and textured-2) exhibit relatively lower friction coefficients than their bare counter parts, especially under low speed and heavy load conditions. The minimum friction coefficient of bare, texture-1 and texture-2 specimens are 0.0116, 0.0095, and 0.0078, respectively. The friction coefficients of texture-2 are the lowest among the three different washers, which may provide evidence for the favorable tribological behavior of the proposed texture design in Section 2.

Moreover, it should be noticed that the minimum friction coefficient for texture-2 occurs at the rotational speed of 240 rpm, obviously lower than that of bare surface (300 rpm) and texture-1 (270 rpm). The arrangement of texture-2 achieves its minimum friction coefficient at the lowest rotational speed among the three gear washers.

As elucidated in Figure 3 in Section 2, the generation of hydrodynamic pressure of partial textured surface may be responsible for the reduction in friction coefficient. Hydrodynamic pressure of micro-textures brings in significant hydrodynamic uplift, which can share part of the load and increase the film thickness. In this case, the contact probability of asperities is reduced, resulting in the increase in friction coefficient. Thus, the lowest point of the Stribeck curve is moved towards the lower left by the hydrodynamic effect of textures, implying the minimum friction coefficient may occur at a relatively low rotational speed for textured washers. Generally, when the rotational speed is higher than a critical value, the uplift caused by hydrodynamic pressure may separate the counter-mating surfaces completely. In this case, the friction is mainly caused by shear resistance of the lubricant. This may be the main mechanism for the relatively lower friction coefficient of textured surface than bare surface in low rotational speed, while in high rotational speed, the tribological improvement effect of surface texturing is insignificant (as shown in Figure 11a–c).

**Figure 12:**
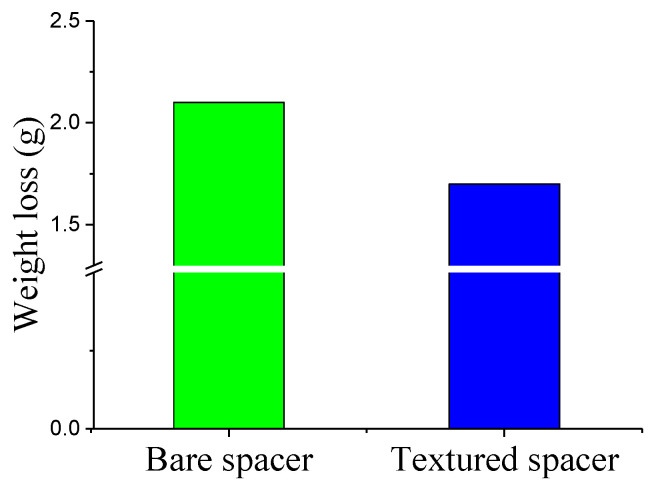
Weight loss of bare and textured gear washers.

In addition, the degree of wear is evaluated by the method of weight loss. Figure 12 illustrates the weight loss for bare and textured-2 washers after the 8 h test (240 rpm, 1450 N). The weight loss of the washer with obtained texture arrangement is obviously lower than that of the bare washer. The reduction rate is about 189%. Hence, the anti-wear effect of the textured gear washer is confirmed.

## 5. Conclusions

This study explored the influence of distributional parameters of textures on tribological performance of gear washers and obtained a preferable surface texturing arrangement according to the mixed lubrication model. Subsequently, corresponding experimental tests were conducted to plot the Stribeck curve of the textured and bare gear washers to facilitate the verification of tribological improvement effect of the optimal design. The main findings of the investigation are given as follows:

1. An annular gear washer partially covered by surface textures yields lower friction coefficients than its bare counterpart;
2. Textures with a sector angle of 20°, texture coverage angle of 12°, circumferential number of 8, and radial number of 6 are selected as the final surface texture distribution arrangement;
3. A washer with the obtained texture arrangement exhibits preferable tribological performance and moves its Stribeck curve towards the lower left, implying significant hydrodynamic pressure effect under low rotational speed.
4. Comparing with the bare washer, the obtained texture arrangement can reduce weight loss caused by wear.

## Figures and Tables

**Table 1:** Coverage area ratio of textures with different *M* and *N*.

	*M*	4	5	6	7	8	9
*N*							
6		7.2%	9%	10.8%	12.6%	14.4%	16.2%
8		9.8%	12.3%	14.8%	17.2%	19.7%	22.1%
10		12.5%	15.6%	18.7%	21.8%	24.9%	28.1%

## Data Availability

Data sharing is not applicable for this article.
